# A Lip Mass Revealing Syphilis: A Case Report With a Review of the Literature

**DOI:** 10.1155/crid/2125721

**Published:** 2026-10-19

**Authors:** May Ahmed Alsenani, Rana Saud Alshagroud, Emad Hadlaq, Bader Mutrik Aldawsari, Hassan Ibrahim Khormi, Abdulrahman A. Altariqi, Norah Ameen Aloushan, Abdulaziz khalid Aloshaywi, Mohammed Abdulaziz Alangari

**Affiliations:** ^1^ Department of Oral Medicine and Diagnostic Sciences College of Dentistry King Saud University Riyadh Saudi Arabia ksu.edu.sa; ^2^ Maxillofacial Surgery and Rehabilitation Department King Fahad Medical City Riyadh Saudi Arabia kfmc.med.sa

## Abstract

**Background:**

Syphilis is a chronic systemic infection caused by *Treponema pallidum* and is well known for its wide spectrum of clinical presentations. Oral manifestations are uncommon and often mimic benign or reactive lesions, making diagnosis challenging, particularly in the absence of overt systemic symptoms.

**Case Presentation:**

We report a case of a 26‐year‐old male who presented with a nonpainful swelling of the lower lip that had persisted for 2 months, with a differential diagnosis including both reactive and neoplastic processes, and underwent a biopsy for definitive diagnosis. Histopathological examination demonstrated ulcerated oral mucosa with psoriasiform epithelial hyperplasia, neutrophilic transmigration, and a dense chronic inflammatory infiltrate rich in plasma cells. Immunohistochemical staining highlighted *Treponema pallidum* organisms within the lesional tissue, and serologic testing was positive, confirming the diagnosis of syphilis. At follow‐up, the patient developed additional oral ulcers and a maculopapular rash involving the palms, soles, and genitalia, consistent with systemic disease, and was referred for medical management.

**Conclusion:**

This case underscores the importance of considering syphilis in the differential diagnosis of atypical oral lesions. Early recognition by dental practitioners, supported by histopathological evaluation, immunohistochemistry, and serologic testing, is essential for timely diagnosis, appropriate referral, and prevention of disease progression and transmission.

## 1. Introduction

Syphilis caused by *Treponema pallidum* [1], a Gram‐negative bacterium that is primarily transmitted through sexual contact, but it can also spread via blood transfusion, direct contact with infectious lesions, and contaminated objects [1]. Additionally, the disease can be congenital, occurring through vertical transmission from an infected pregnant woman to her fetus via transplacental route [2]. Following the introduction of penicillin and other antibiotics, the prevalence of the disease declined; however, syphilis has never been entirely eliminated [3]. The recent rise in syphilis cases can be attributed to an increase in intravenous drug use, a reduction in safe sex practices, and a higher number of sexual partners [4]. Syphilis progresses through several stages in its natural course: primary, secondary, latent (asymptomatic), and tertiary [5–7]. Once the *Treponema pallidum* bacterium enters the human body, it rapidly spreads through the bloodstream and lymph nodes [4]. The first lesion, called a chancre, appears 10–90 days after exposure as a painless ulcer at the infection site [4]. The secondary stage is marked by various skin and oral lesions, along with systemic symptoms, such as fatigue, weight loss, headaches, fever, muscle aches, and joint pain [5]. This stage has a wide range of clinical manifestations that can resemble other diseases [8]. In the latent phase, patients do not show symptoms or lesions but still test positive on serological tests [4]. Tertiary syphilis occurs in about one‐third of untreated cases, with the most common oral lesion being a syphilitic gumma, an ulcerated, nodular, and painless lesion that can cause tissue destruction and perforation of the palate [1]. Syphilis has historically been called the “great imitator” because its diverse presentations can mimic other conditions [7]. These diverse manifestations can complicate diagnosis for clinicians and pathologists, potentially resulting in delayed or incorrect diagnosis [1]. Timely identification of the histopathologic and clinical features of oral syphilis is crucial for accurate diagnosis, better patient outcomes, and effective disease prevention [1]. Herein, we present a unique case of syphilis, accompanied by a literature review.

## 2. Case Presentation

A 26‐year‐old unmarried male presented to the Oral Medicine Clinic with a chief complaint of “swelling in my lower lip” that had persisted for approximately 2 months. The patient reported no associated pain. Upon clinical examination, he was alert and oriented, with no palpable cervical lymphadenopathy. Extraoral examination revealed a well‐defined, firm swelling of the lower lip. The lesion was nontender and exhibited infrequent bleeding, with no overlying ulceration or purulent discharge. Intraoral examination revealed a solitary, localized lesion involving the lower labial mucosa, without extension beyond the mucosal surface or involvement of deeper anatomical structures (Figure 1A). No other intraoral abnormalities were identified. Radiographic evaluation, including panoramic and occlusal views, revealed no bony involvement or other pathological abnormalities.

**Figure 1:**
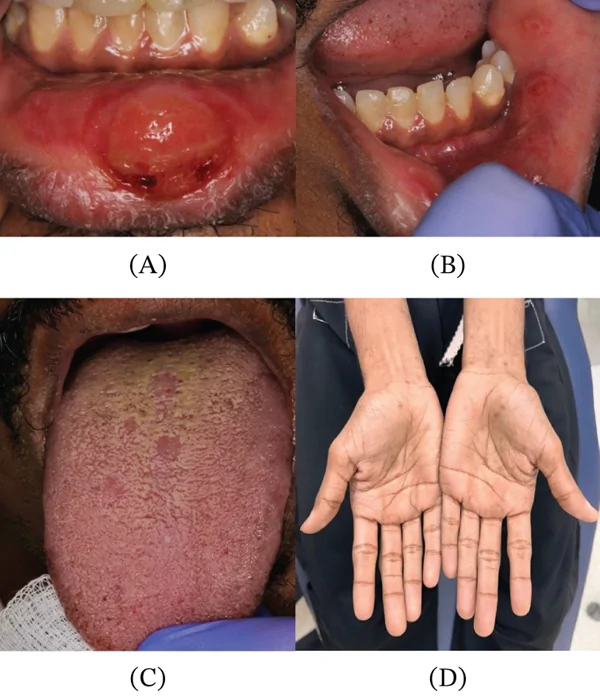
(A) Ulcerated mass measuring approximately 1.5 × 1.5 cm on the lower labial mucosa. (B) Multiple aphthous‐like ulcers on the buccal mucosa. (C) Indurated and slightly papillary nodules on the dorsal tongue. (D) A maculopapular cutaneous rash involving the palms.

The differential diagnosis included both reactive and neoplastic processes. Therefore, an excisional biopsy was planned and performed under local anesthesia, and the specimen was submitted for histopathological evaluation.

Histopathological examination revealed multiple sections of ulcerated oral mucosa associated with a dense chronic inflammatory infiltrate. At the periphery of the ulcer, the epithelium exhibited a thick keratinous surface crust admixed with inflammatory cells, focal psoriasiform epithelial hyperplasia, neutrophilic transmigration, spongiotic pustule formation, and reactive epithelial atypia. The inflammatory infiltrate was prominent and arranged in a vaguely lobular pattern, extending from the epithelial–connective tissue interface into the deeper tissues. It involved adjacent blood vessels, nerve fibers, and skeletal muscle bundles, resulting in focal muscle fiber degeneration. Extension into salivary gland lobules was also observed, accompanied by squamous metaplasia and acinar atrophy. The inflammatory infiltrate was composed predominantly of plasma cells, many showing binucleation or multinucleation with rare mitotic figures, admixed with lymphocytes, epithelioid histiocytes, and scattered eosinophils. Occasional large, atypical cells with round‐to‐ovoid irregular nuclei, some exhibiting binucleation and vesicular nucleoli, were also identified. The histopathological findings raised suspicion for syphilis, and further ancillary investigations were performed. Periodic acid–Schiff (PAS) and Grocott methenamine silver (GMS) stains were negative for fungal organisms. Immunohistochemical staining for kappa and lambda light chains demonstrated a polyclonal plasma cell population. Treponema pallidum immunohistochemistry (IHC) demonstrated dense positivity within the lower basal and spinous epithelial layers, with additional perivascular distribution, supporting the diagnosis of syphilis. Additionally, Verhoeff–Van Gieson stain highlights the vascular architecture, with focal subtle vascular wall changes but no definite significant intimal thickening or luminal narrowing (Figure 2A–D).

**Figure 2:**
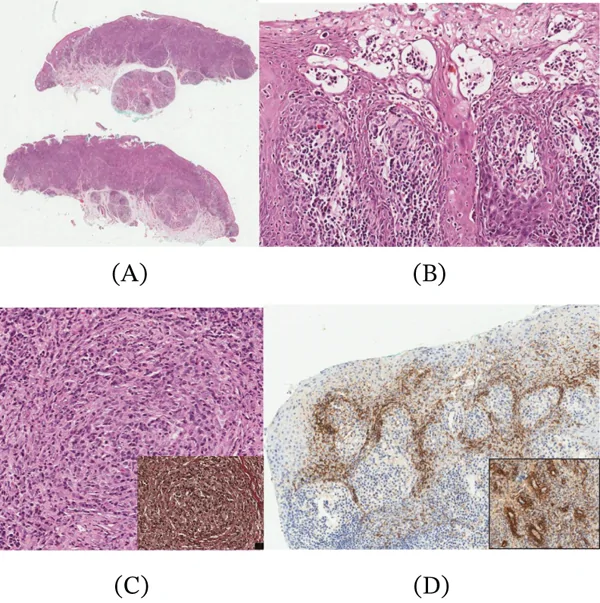
(A) Low‐power view of ulcerated oral mucosa demonstrating a lobular pattern of dense chronic inflammatory infiltrate. (B) Extensive neutrophilic exocytosis and spongiotic pustule formation. (C) Prominent plasma cell–rich inflammatory infiltrate with lobular and storiform architecture resulting in vascular compression, Verhoeff–Van Gieson stain highlights the vascular architecture, with focal subtle vascular wall changes but no definite significant intimal thickening or luminal narrowing. (inset). (D) *Treponema pallidum* immunohistochemistry showing dense positivity in the lower basal and spinous epithelial layers and perivascular distribution (inset).

Serologic testing further confirmed active infection. The initial treponemal screening assay was positive, with a cutoff index (COI) of 180.0. Confirmatory testing with Treponema pallidum hemagglutination assay (TPHA) was positive at a titer of 1:2048, and the rapid plasma reagin (RPR) test was reactive at a titer of 1:64. Taken together, the clinical, histopathological, immunohistochemical, and serologic findings confirmed the diagnosis of primary syphilis. At follow‐up, the patient developed additional oral ulcers and a maculopapular rash involving the palms, soles, and genital region, consistent with progression to secondary syphilis (Figure 1B–D). The patient was referred to the Infectious Diseases Department for treatment, further evaluation, and management.

## 3. Materials and Methods

A literature review was conducted using PubMed, ResearchGate, and Google Scholar to identify published reports of syphilis presenting with oral manifestations. Articles published between January 2015 and January 2025 were included, with the search restricted to case reports and case series. Search terms were used both individually and in combination, including oral ulcer, infection, gumma, chancre, oral manifestations of syphilis, mucous patch, and *Treponema pallidum*. Additionally, the reference lists of all relevant articles were manually reviewed to identify any additional eligible cases.

The initial search yielded 75 articles. Studies were excluded if they were published in languages other than English or lacked essential clinical or demographic information, including patient age, sex, geographic region, lesion location, or stage of disease. After applying the eligibility criteria, 60 articles were included in the final analysis. Data extracted from each case included patient age and sex, geographic distribution, lesion location, site involvement (single or multiple sites), and stage of disease.

## 4. Results

A total of 130 patients with syphilis presenting with oral manifestations were identified from the reviewed literature. The demographic and clinical characteristics of the included cases are summarized in Table 1.

**Table 1:** Demographic and clinical characteristics of reported cases of syphilis with oral manifestations.

Category	Variable	*n*	%
Gender	Male	78	60.0
	Female	52	40.0
Age (years)	Mean (median)	35.9 (34)	—
	Range	9‐83	—
Age distribution	1st decade (0–9)	1	0.8
	2nd decade (10–19)	14	10.8
	3rd decade (20–29)	38	29.2
	4th decade (30–39)	28	21.5
	5th decade (40–49)	25	19.2
	6th decade (50–59)	16	12.3
	7th decade (60–69)	6	4.6
	8th decade (70–79)	0	0.0
	9th decade (80–89)	2	1.5
Geographic distribution	Brazil	78	60.0
	USA	12	9.2
	Argentina	8	6.2
	China	7	5.4
	South Africa	4	3.1
	Spain	3	2.3
	Italy	3	2.3
	Japan	3	2.3
	India	3	2.3
	Mexico	2	1.5
	UK	2	1.5
	Canada	1	0.8
	Portugal	1	0.8
	Kuwait	1	0.8
	Poland	1	0.8
	Turkey	1	0.8
Oral site involvement	Tongue	73	56.2
	Lower lip	53	40.8
	Upper lip	41	31.5
	Palate	32	24.6
	Buccal mucosa	25	19.2
	Lip commissure	19	14.6
	Oropharynx	17	13.1
	Gingiva	6	4.6
	Retromolar area	4	3.1
Number of oral sites	Single site	58	44.6
	Multiple sites	72	55.4
Stage of syphilis	Primary	26	20.0
	Secondary	102	78.5
	Tertiary	2	1.5
Clinical presentation	Mucous patch	79	60.8
	Ulcer	72	55.4
	Nodule	11	8.5
	Split papules	10	7.7
	Papillary nodules	5	3.8

The study population was predominantly male accounted for 60.0% (*n* = 78), while females accounted for 40.0% (*n* = 52). The mean age at presentation was 35.9 years (median: 34 years), with an age range of 9–83 years. Most cases occurred in the third decade of life (29.2%), followed by the fourth (21.5%) and fifth decades (19.2%).

Regarding geographic distribution, the majority of cases were reported from Brazil (*n* = 78, 60.0%), followed by the United States (*n* = 12, 9.2%), Argentina (*n* = 8, 6.2%), and China (*n* = 7, 5.4%). Other countries each contributed less than 5% of the total cases.

Analysis of oral site involvement revealed that the tongue was the most frequently affected site (*n* = 73, 56.2%), followed by the lower lip (*n* = 53, 40.8%), upper lip (*n* = 41, 31.5%), and palate (*n* = 32, 24.6%). Less commonly involved sites included the buccal mucosa (19.2%), lip commissure (14.6%), oropharynx (13.1%), gingiva (4.6%), and retromolar area (3.1%). Multiple oral sites were involved in 72 patients (55.4%), whereas 58 patients (44.6%) presented with a single oral lesion site.

With respect to disease stage, secondary syphilis was the most frequently reported stage (*n* = 102, 78.5%), followed by primary syphilis (*n* = 26, 20.0%), and only two cases (1.5%) were classified as tertiary syphilis.

Clinically, the most common oral presentations were mucous patches (*n* = 79, 60.8%) and ulcerative lesions (*n* = 72, 55.4%). Less frequent clinical features included nodules (8.5%), split papules (7.7%), and papillary nodules (3.8%).

## 5. Discussion

Recognizing the oral manifestations of syphilis is crucial for both clinicians and pathologists, as failure to do so may lead to misdiagnosis and serious consequences for the patient [9]. The clinical presentation of the disease is highly dependent on the stage of infection [10]. In our review, males were more frequently affected than females. Most patients were in their third and fourth decades of life. Geographically, cases were reported internationally, but the majority originated from Brazil. The tongue, followed by the lower lip, were the most involved oral sites, and many patients demonstrated multi‐site involvement rather than a single localized lesion. Most individuals presented in the secondary stage of syphilis.

In terms of clinical presentation, mucous patches and ulcerative lesions were the most frequently observed oral manifestations in the present review, accounting for 60.8% and 55.4% of cases, respectively. These findings are largely compatible with previous studies, which consistently identify mucous patches and ulcers as the predominant oral manifestations of syphilis, particularly in the secondary stage. Thums et al. [10] reported findings consistent with ours and noted varied clinical presentations, with 33% of cases showing erosive/ulcerative lesions and the majority, 85%, showing grayish‐white plaques [10]. In contrast, Matias et al. [11]. analyzed 85 medical records of patients with oral syphilis and found that only 20% of lesions were mucous plaques, with most lesions appearing as ulcerated [11]. This highlights the variability in oral syphilis manifestations across different studies. In our review, 55.4% of the cases exhibited multiple site involvement, and 78.5% were identified as being in the secondary stage of syphilis. This observation is consistent with existing literature, which indicates that oral lesions in secondary syphilis are often multicentric [7, 10, 12].

In the present case, the clinical differential diagnosis for an ulcerated mass in the lower lip encompasses a wide range of possibilities, including benign reactive conditions, infectious diseases, and malignant neoplasms.

Among benign reactive conditions, the differential includes traumatic ulcer, traumatic ulcerative granuloma with stromal eosinophilia (TUGSE), and necrotizing sialometaplasia (NS). Traumatic ulcer and TUGSE may resemble the clinical presentation in this case, as they typically arise at sites subjected to local irritation or trauma [13]. However, these entities are less favored here due to the absence of an identifiable traumatic source. Necrotizing sialometaplasia is a benign, self‐limiting inflammatory condition caused by ischemic infarction of salivary gland tissue [14]. It usually begins as a nonulcerated swelling, sometimes associated with pain or paresthesia, and subsequently evolves into a crater‐like ulcer after mucosal sloughing [14]. Although the posterior hard palate is the most frequently affected site, NS has been reported in other locations containing minor salivary glands and can theoretically occur wherever such tissue is present [15–17]. Recognized risk factors include the administration of a greater palatine nerve block, Raynaud disease, Buerger disease, bulimia, excessive tobacco or alcohol use, local trauma, anabolic steroid abuse, and proximity to malignancy [14]. In this case, NS was considered due to the lesion’s clinical features and the patient’s smoking history. However, the diagnosis appeared less likely given the atypical location (lower lip), the absence of a clear ischemic trigger, and the persistence of the lesion for 2 months without spontaneous resolution.

Tuberculosis (TB) is a chronic infectious disease caused by *Mycobacterium tuberculosis*, typically affecting the lungs but capable of involving virtually any organ system, including the oral cavity [18, 19]. Oral TB is rare and most often occurs as a secondary manifestation of pulmonary disease [18, 19]. It is usually accompanied by systemic symptoms such as productive cough, fever, night sweats, malaise, and weight loss [18, 19]. TB was considered in this case due to the chronicity of the lesion [18, 19]. Nevertheless, the absence of systemic symptoms, no reported history of active or latent TB, and the patient’s young, immunocompetent status reduced the likelihood of this diagnosis.

Deep fungal infections, including histoplasmosis, blastomycosis, and paracoccidioidomycosis, can clinically mimic malignancy and are therefore part of the differential diagnosis. However, such infections are exceedingly rare in immunocompetent individuals without systemic signs, relevant travel or environmental exposures, or immunosuppressive conditions [20–22]. The rarity of isolated oral presentations in otherwise healthy young individuals living in nonendemic regions further decreases the probability of this diagnosis.

Leishmaniasis is a parasitic infection caused by various *Leishmania* species. It is endemic in certain areas of Saudi Arabia and may occasionally present with oral lesions [23–25]. The disease may manifest in cutaneous, mucocutaneous, or visceral forms, depending on the species and host immune status [23]. While oral leishmaniasis has been reported, primary isolated oral involvement is extremely rare, particularly in the absence of accompanying cutaneous or systemic disease [26]. The local species prevalent in Saudi Arabia, *L. major* and *L. tropica*, are more commonly associated with cutaneous form rather than mucosal involvement, making this diagnosis improbable in the present case [25].

Cytomegalovirus (CMV) associated ulcers represent a significant infectious consideration in the differential diagnosis of persistent, indurated oral lesions [27, 28]. While these ulcers can clinically mimic the indurated borders of a syphilitic chancre, they are almost exclusively identified in immunocompromised individuals, including organ transplant recipients, patients receiving systemic immunosuppressive therapy, or those with age‐related immunosenescence [27, 28]. In the context of the current data where the patient population was predominantly younger (median age 34) and immunocompetent, CMV is considered highly unlikely based on our results. However, in cases of nonhealing oral ulcerations with an ambiguous etiology, a diagnostic panel approach utilizing IHC for CMV is recommended to definitively rule out viral‐induced tissue necrosis [1].

EBV‐positive mucocutaneous ulcer (EBVMCU) is a distinct, newly recognized clinicopathological entity that often enters the differential diagnosis for oral syphilis because it typically presents as a well‐circumscribed, isolated ulcer in the oropharyngeal mucosa [27, 28]. Histopathologically, EBVMCU mimics the dense lymphoplasmacytic infiltrate seen in syphilis but is characterized by atypical large B‐cell blasts with Hodgkin/Reed–Sternberg‐like morphology [28]. These lesions are definitively distinguished by strong CD30 and EBER (Epstein–Barr virus‐encoded small RNA) positivity [28]. Furthermore, EBVMCU is primarily associated with various forms of immunosuppression and an older patient demographic (median age 77), which stands in contrast to the clinical profile of the syphilis cases reviewed.

Finally, malignant neoplasms, particularly oral squamous cell carcinoma (OSCC), were considered in the clinical differential diagnosis. OSCC is the most common malignancy of the oral cavity and may present clinically as a persistent indurated ulcer or ulcerated mass with raised or rolled margins and occasional bleeding, features that can closely mimic benign reactive or infectious lesions [29]. However, the patient’s young age and absence of significant risk factors made this diagnosis less likely clinically.

In the present case, microscopic examination revealed multiple sections of ulcerated oral mucosal tissue with dense chronic inflammatory infiltrate. The epithelium at the ulcer periphery exhibited a thick surface keratin crust intermixed with inflammatory cells, focal psoriasiform epithelial hyperplasia with neutrophilic transmigration and spongiotic pustule formation, and reactive atypia. The inflammatory infiltrate was robust and displayed a vaguely lobular configuration, extending from the epithelial–connective tissue interface deep into surrounding structures, including blood vessels, nerve fibers, and skeletal muscle fibers, causing muscle degeneration and extending to salivary gland lobules with squamous metaplasia and acinar atrophy. The inflammatory cells were mainly plasma cells, showing binucleation, multinucleation, and sparse mitotic figures, admixed with lymphocytes, epithelioid histiocytes, scattered eosinophils. Occasional large cells with round‐to‐ovoid irregular nuclei, some with binucleation and vesicular nucleoli, were also noted.

The histopathological differential diagnosis included chronic infections with plasma cell predominance such as syphilitic ulcer and deep fungal infection, plasma cell mucositis, plasma cell neoplasm, and lymphoproliferative disorders rich in plasma cells such as MALT lymphoma.

The presence of psoriasiform epithelial hyperplasia with neutrophilic transmigration and a polyclonal plasma cell infiltrate raised the possibility of plasma cell mucositis [30]. Plasma cell mucositis is generally regarded as a hypersensitivity reaction to contactants such as flavoring agents in chewing gum, candies, toothpaste, or mouthwash [30]. In this case, however, the heterogeneity of the inflammatory infiltrate, which also contained lymphocytes, epithelioid histiocytes, and mast cells, along with the presence of cellular atypia and the patient’s denial of using such products or having a history of allergy, made classic plasma cell mucositis less likely.

Deep fungal infection may present clinically and histologically with ulceration, a mixed chronic inflammatory infiltrate, plasma cell predominance, and granuloma formation [31]. Given these overlapping features, it was initially considered in the differential diagnosis. However, special stains including PAS and GMS were negative for fungal organisms, thereby excluding this possibility.

Plasma cell neoplasm, particularly solitary extramedullary plasmacytoma, represented another important differential diagnosis because of the plasma cell–rich infiltrates, cytological atypia, and the nodular architectural configuration [32]. Nevertheless, the absence of light‐chain restriction in this case effectively argued against such a diagnosis.

The histopathological features also raised the possibility of a mucosa‐associated lymphoid tissue (MALT) lymphoma, given the dense plasma cell‐rich infiltrate with occasional large atypical cells in a background of chronic inflammation [33]. MALT lymphomas are known to arise in sites of chronic antigenic stimulation and frequently display extensive plasma cell differentiation that may mimic reactive or plasmacytic lesions [33]. In addition, the presence of occasional large nucleolated cells could be interpreted as scattered transformed B‐cells, a finding sometimes observed in low‐grade MALT lymphomas.

However, several features in this case argued against MALT lymphoma. First, the infiltrate was overwhelmingly composed of mature plasma cells with only scattered lymphocytes, whereas MALT lymphomas are typically dominated by small centrocyte‐like B‐cells and monocytoid B‐cells, often with associated lymphoepithelial lesions [33]. In this specimen, no lymphoepithelial lesions or destructive lymphoid infiltrates of ductal epithelium were identified. Second, the overall architecture showed a vaguely lobular and storiform plasma cell infiltrate rather than the diffuse, monotonous B‐cell proliferation characteristic of MALT lymphoma. Finally, IHC confirmed a polyclonal plasma cell population without light‐chain restriction. This finding strongly supports a reactive rather than a neoplastic process. The clinical presentation of an ulcerated mass, together with histopathological features of psoriasiform epithelial hyperplasia with neutrophilic transmigration and a polyclonal plasma cell infiltrate, raised the possibility of syphilis. This prompted serologic and immunohistochemical testing, both of which were positive, confirming the diagnosis. IHC is considered more sensitive than Warthin–Starry staining for the detection of *Treponema pallidum* and may aid in distinguishing syphilitic infection from other oral spirochetal organisms, particularly *Treponema denticola* [1]. Histologically, IHC highlights numerous corkscrew‐shaped spirochetes infiltrating the epithelium, occasionally demonstrating a perivascular distribution within the underlying fibrous stroma [1]. Martín‐Ezquerra et al. reported that *Treponema pallidum* demonstrates distinct distribution patterns in primary and secondary syphilis identifiable by IHC, with secondary lesions showing a predominantly epitheliotropic pattern, whereas primary lesions additionally exhibit a perivascular vasculotropic distribution, similar to the findings observed in our case [34]. However, these findings have not been extensively studied in the literature. In addition, cross‐reactivity of anti‐*Treponema pallidum* antibodies with other bacterial organisms, including *Borrelia burgdorferi* and members of the *Brachyspira* family, has been documented [1]. Therefore, careful clinicopathologic correlation remains essential for accurate diagnosis. First‐line treatment consists of benzathine penicillin G, while doxycycline or azithromycin may be considered alternative therapeutic options in patients with penicillin allergy [35].

## 6. Conclusion

Syphilis remains a diagnostic challenge due to its diverse and often deceptive clinical presentations, particularly when it manifests in the oral cavity. This case highlights an unusual presentation of syphilis as a localized lower lip swelling, initially mimicking common benign oral lesions. The integration of clinical findings, histopathological features, immunohistochemical detection of *Treponema pallidum*, and serologic confirmation was critical in establishing the correct diagnosis.

Dental professionals play a pivotal role in the early detection of syphilis, as oral lesions may represent the first or only manifestation of the disease. Awareness of this possibility and a high index of suspicion are essential to ensure prompt diagnosis, appropriate referral, and effective management, thereby reducing the risk of systemic complications and further transmission.

## Funding

No funding was received for this manuscript.

## Conflicts of Interest

The authors declare no conflicts of interest.

## Data Availability

The data that support the findings of this study are available from the corresponding author upon reasonable request.
